# Preliminary assessment of the potential role of urbanization in the distribution of carbamate and organophosphate resistant populations of *Culex* species in Ghana

**DOI:** 10.1186/s13071-014-0621-4

**Published:** 2015-01-08

**Authors:** Andreas A Kudom, Ben A Mensah, Guenter Froeschl, Daniel Boakye, Heinz Rinder

**Affiliations:** Center for International Health, Department of Infectious Diseases and Tropical Medicine, Ludwig-Maximilians-Universität Munich, Munich, Germany; Department of Entomology and Wildlife, School of Biological Sciences, University of Cape Coast, Cape Coast, Ghana; Noguchi Memorial Institute for Medical Research, University of Ghana, P.O. Box LG 581, Legon, Ghana; Bayerisches Landesamt für Gesundheit und Lebensmittelsicherheit (LGL), Veterinärstraße 2, Oberschleißheim, 85762 Germany

**Keywords:** *ace*1, Carbamate, *Culex decens*, *Culex quinquefasciatus*, Ecology, Esterase, Organophosphate, Insecticide-resistance, Urbanization

## Abstract

**Background:**

Besides its role as a pathogen vector, *Culex* species also indirectly promotes the transmission of malaria if the use of bed nets or indoor residual spraying is discontinued due to a lack of insecticide efficacy against it. A recent survey revealed widespread occurrence of pyrethroid resistance among urban populations of this mosquito in Ghana. In order to plan and implement insecticide-based resistance management strategies, this study was carried out to assess resistance status of *Culex* species to organophosphate and carbamate in urban areas in Ghana and the possible mechanisms involved as well as environmental factors associated with its distribution.

**Methods:**

Mosquito larvae were sampled from various land use and ecological settings and in different seasons. In adults, susceptibility to organophosphates (fenitrothion, malathion) and carbamates (propoxur, bendiocarb) were determined. Mixed function oxidase (MFO) and α- and β-esterase assays, as well as a PCR diagnostic assay to determine *ace*1 mutation were performed in individual mosquitoes.

**Results:**

*Culex quinquefasciatus* as well as *C. decens* and other unidentified *Culex* species were found breeding in polluted water bodies in the study sites. Across all sites and seasons, carbamate induced mortality was 94.1% ± 15.4 whereas mortality caused by organophosphate was 99.5% ± 2.2. In addition, *ace*1 mutation and high levels of esterases were detected in some of the mosquito populations. There was a strong correlation between susceptibility status of the mosquitoes and the level of absorbance of β-esterase (Pearson r = − 0.841, p = 0.004).

**Conclusions:**

The study found low prevalence of resistance to carbamate and organophosphate insecticides among *Culex* species from Ghana. However, there were populations with *ace*1 mutations and high levels of esterases, which can confer high resistance to these classes of insecticides. Thus, it is important to monitor activities or behaviour that has the potential to select for carbamate and organophosphate resistance populations.

## Background

Recommended options for malaria control include the use of long-lasting insecticide-treated bed nets (LLIN) and indoor residual spraying (IRS) [1]. Unfortunately, LLIN is highly dependent on a single class of insecticides, the pyrethroids, for which malaria vectors and other mosquitoes have developed resistance. In order to act before insecticide resistance compromises current vector control strategies, WHO has proposed various guidelines to encourage countries to plan and implement insecticide resistance management strategies [2].

Successful resistance management depends upon reducing the selection pressure exerted by a particular insecticide or a particular mode of action [3]. Four classes of insecticides, namely pyrethroids, organochlorine (exclusively DDT), organophosphates and carbamates are used widely in the public health sector; however, pyrethroids and DDT share similar modes of action, thus making organophosphate and carbamate very important in resistance management strategies. Various resistance management strategies including rotations, mosaics and mixture of pyrethroid and/or organophosphate or carbamate for IRS and on nets have been demonstrated both in laboratory and field conditions [4,5]. As a result, knowledge on resistance status of vectors against organophosphate or carbamate and the mechanism involved as well as factors that influence the resistance have become important.

Until recently, most research efforts on insecticide resistance in mosquitoes have been focused on determining the mosquitoes’ resistance status and the mechanisms responsible. Less attention has been paid to the impact of environmental factors that can influence resistance in mosquitoes. Recent studies have found pollutants in urban water bodies, which are mostly generated by motor vehicles, industries and domestic waste, to affect mosquito detoxification enzymes leading to enhanced insecticide degradation [6]. Several studies in Ghana have shown different levels of susceptibility to organophosphates and carbamates in *Anopheles* mosquitoes including the presence of *ace*1 mutation in some populations [7-9]. However, for *Culex* species, which also plays a critical role in malaria control, information on susceptibility to organophosphate and carbamate is limited. Besides its role as a pathogen vector itself, *Culex* species also indirectly promotes the transmission of malaria, if the use of bed nets or IRS is discontinued due to a lack of insecticide efficacy against it. A recent survey revealed widespread occurrence of pyrethroid resistance among urban populations of this mosquito in Ghana (Kudom et al. unpublished observations.). This study was therefore carried out to assess resistance status of *Culex* species to organophosphate and carbamate in urban areas in Ghana and the possible mechanisms involved as well as environmental factors associated with its distribution.

## Methods

### Study sites

**Figure 1:**
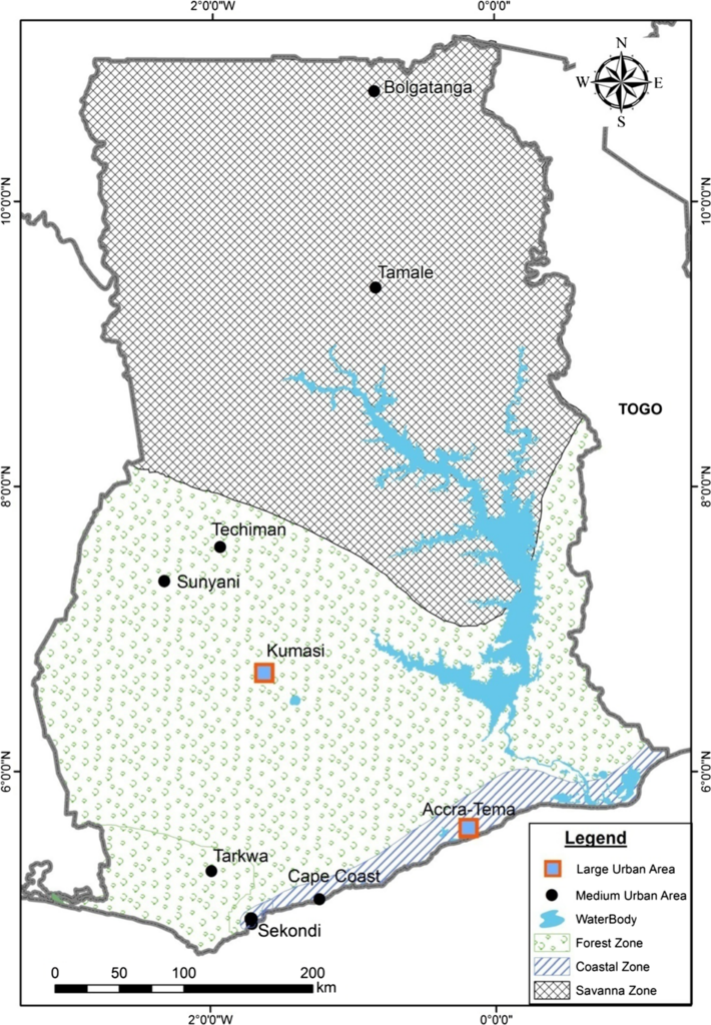
**Map of Ghana showing the three ecological zones and urban towns where mosquitoes were sampled.**

Mosquito larvae were collected from ponds, polluted drains and choked gutters in nine urban areas in Ghana (Figure 1). Urban areas were selected according to ecological settings and in each selected town, mosquitoes were sampled from different land use settings; residential, urban agricultural and swampy areas. Mosquito larvae were also sampled in both rainy and dry seasons. Larvae were brought to the laboratory for emergence and testing of adults.

### Susceptibility test

An adult susceptibility assay was carried out using WHO discriminating dosages of two insecticides each from organophosphate (Fenitrothion 1%, Malathion 5%) and carbamate (Bendiocarb 0.1%, Propoxur 0.1%). Mosquitoes were exposed to the insecticides according to WHO guidelines (WHO/VBC/81.806). Mortality resulting from tarsal contact with treated filter paper was measured using WHO test kits. Four batches of 20–25 unfed females, aged 2–4 days, were exposed to papers impregnated with malathion or bendiocarb for 1 h, fenitrothion or propoxur for 2 h.

### Biochemical assay

Mixed function oxidase (MFO) and α- and β-esterase were assayed in individual 2–4 day old frozen (−80°C) adults that had been reared from larvae and not been previously exposed to insecticides in the laboratory. The procedures in preparing the solutions and conducting the experiments followed the method described by Hemingway [10]. A total of about 450 mosquitoes, comprising fifty female individuals from each of the nine study sites were used for the biochemical assay. Mosquitoes were homogenized in potassium phosphate buffer (pH 7.2) and the homogenate was loaded into micro plate wells in duplicate on the same plate for each enzyme assay. A plate-reading spectrophotometer was used to collect data at the appropriate absorbing wavelength (nm).

For the esterase assay, 100 μl mosquito homogenate with 100 μl α- or β-naphthyl acetate was incubated at room temperature for 10 minutes. Then, 100 μl dianisidine solution was added to the mixture and incubated for 2 minutes. The plate was then read using a 620 nm filter for α-naphthyl and 540 nm filter for β-naphthyl.

For the oxidase assay, 100 μl mosquito homogenate with 200 μl of Tetramethyl-Benzidine and 25 μl of 3% hydrogen peroxide were incubated for 5 minutes. The plate was then read using a 620 nm filter.

### Species identification and detection of *ace*1 mutations

Genomic DNA was extracted from 20 mosquitoes each from the nine urban sites. These mosquitoes had not been previously exposed to insecticide. The DNA was extracted with DNeasy extraction kit (QIAGEN) based on the protocol from the manufacturer.

Mosquitoes for this study were mostly collected from polluted water bodies; hence *C. quinquefasciatus* was generally expected. PCR diagnostic assay was therefore carried out to identify *C. quinquefasciatus* using the method described by Smith and Fonseca [11]. Four primers (“ACEquin”,“B1246s”, F1457” and “B1246) (Table 1) were used to detect *C. quinquefasciatus* and any other species that belongs to the *C. pipiens* complex.

**Table 1:** **Oligonucleotides (primers) used for identification of** ***Culex*** **species and detection of** ***ace*** **1 mutation**

**Type of molecular assay**	**Primers**
Species identification	ACEquin 5’-CCTTCTTGAATGGCTGTGGCA-3’
	B1246s 5’-TGGAGCCTCCTCTTCACGG-3’
	F1457 5’-GAGGAGATGTGGAATCCCAA-3’
	B1246 5’-TGGAGCCTCCTCTTCACGGC-3’
	Cddir 5’-ACCTCGACGATACTCCGATTT-3’
	Cdrev 5’-TGTGTTCTGCAGGAGGAAGA-3’
	LCO1490 5’-GGTCAACAAATCATAAAGATATTGG-3’
	HCO2198 5’-TAAACTTCAGGGTGACCAAAAAATCA-3’
Detection of *ace*1 mutation	Moustdir1 5’-CCGGGNGCSACYATGTGGAA-3’
	Moustrev1 5’-ACGATMACGTTCTCYTCCGA-3’

Also, the universal DNA primers, LCO1490 and HCO2198 (Table 1) of Folmer *et al*. [12] were used to amplify an 830 bp region of the mitochondrial cytochrome oxidase subunit I gene of four mosquitoes randomly selected from the mosquitoes that failed to amplify from the previous PCR assays.

Based on the results from the sequence, two primers (Cddir and Cdrev) (Table 1) were designed from *C. decens* cytochrome c oxidase subunit 1 (CO1), which was obtained from Genebank with accession number AY64524. The primer was designed with Primer 3® software. PCR was conducted with Cddir and Cdrev on the DNAs that failed to amplify in the previous PCR assay.

Each PCR contained 5 ml of 10 PCR buffer, 1.5 mM of MgCl_2_, 35 ml of distilled water, 200 mM of each dNTP, 1 unit of Taq polymerase, 0.3 mM of each primer and 3 μl of DNA template. The PCR thermal condition consisted of one cycle of 1 min at 94°C; five cycles of 1 min at 94°C, 1.5 min at 45°C and 1.5 min at 72°C; 35 cycles of 1 min at 94°C, 1.5 min at 50°C and 1 min at 72°C and a final cycle of 5 min at 72°C. To confirm a successful reaction, a 7 μl sample from each reaction was then run via electrophoresis through a 2% agarose gel with ethidium bromide and visualized using ultraviolet (UV) light.

The four PCR products amplified by the universal DNA primers were purified using a QIAGEN QIAquick® PCR purification kit according to the manufacturers protocol and sequenced in both forward and reverse directions on an ABI 377 automated sequencer (Applied Biosystems) using the Big Dye v. 3 sequencing kit.

PCR-RFLP diagnostic test to detect *ace*1 mutation were carried out on the extracted DNA from the nine study sites. In the PCR assay, 3 μl of genomic DNA was amplified with the primers “Moustidir1” and “Moustrev1” (Table 1) described by Weill et al. [13]. PCR was conducted in 25 μl volumes containing 1× PCR buffer containing 1.5 mM MgCl_2_, 0.2 mM of each dNTP, 3.1 μl of each of the primers, one unit of Taq polymerase (HotstarTaq: Qiagen®). The PCR conditions included an initial denaturation step at 95°C for 15 min followed by thirty five cycles of 94°C for 30 s, 54°C for 30 s, and 72°C for 30 s, and a final extension at 72°C for 5 min.

Fifteen μl of the PCR product were digested with 5 units of *Alu*1 restriction enzyme in the final volume of 20 μl and incubated at 37°C for 16 h. The restriction products were fractionated on a 2% agarose gel and visualised by ethidium bromide staining under UV light.

### Statistical analysis

Percentage mortality was calculated from the results of the bioassay. Percentage mortalities from different land use and ecological zones were compared using Kruskal-Wallis test whereas difference in mortalities between the two classes of insecticides and seasons were compared using Mann Whitney test. Study sites were categorized into large urban area (metropolitan area with several sub-metro and human population more than one million) and small urban area (a metropolitan or municipal area with human population less than one million) as well as percentage mortality into resistant (mortality less than 98%) and susceptible (mortality more than 98%) based on WHO [14] criteria. Pearson’s Chi-square and correlation test was used to determine the association between percentage mortality and enzyme levels or resistance status and urban size. Results from the esterase assays were compared between study sites with ANOVA (Analysis of Variance) and post hoc test using Fisher’s Least Significant Difference. All the tests were done with SPSS® (version 20). Phylogenetic tree was constructed using Mega 6 software from aligned DNA sequences of mitochondrial cytochrome c oxidase (CO1) gene using 500 boot-straps.

## Results and discussion

### Distribution of *Culex* species

**Figure 2:**
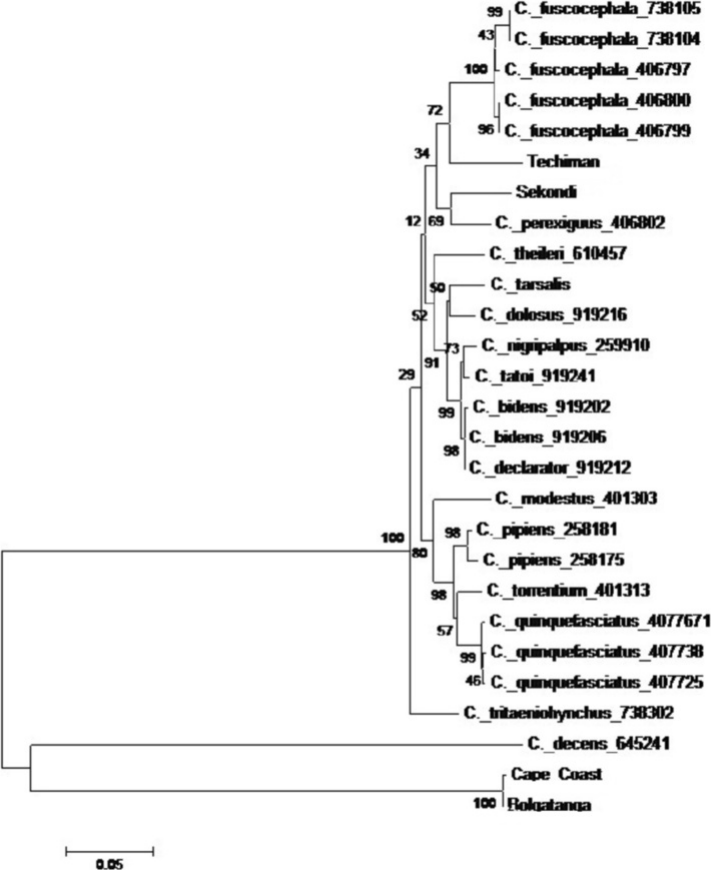
**Phylogenetic tree constructed from aligned DNA sequences of mitochondrial cytochrome c oxidase (CO1) gene using 500 boot-straps with Mega 6 software.** Techiman, Sekondi, Cape Coast and Bolgatanga are the samples collected in this study.

*Culex quinquefasciatus* was generally expected due to the breeding habitat in which the mosquitoes were collected. Nevertheless, PCR diagnostic assay confirms *C. quinquefasciatus* in only Accra and Kumasi. Besides *C. quinquefasciatus*, no member of the *C. pipiens* complex was found. Data from mitochondrial COI gene sequence did not match 100% with any existing COI sequence in GeneBank. The phylogenetic analysis (Figure 2) indicated that the unidentified *Culex* species could be made up of two or more different species. From the branches of the phylogenetic tree, it could be inferred that *Culex* species from Bolgatanga and Cape Coast were either *C. decens* or closely related to it whereas *Culex* species from Techiman and Sekondi were closely related to *C. fuscocephala* or *C. perexiguus.* The genetic resemblance of the *Cule*x species from Techiman and Sekondi to *C. fuscocephala* and *C. perexiguus* was unexpected since such species have not been reported in Ghana before. Further studies, combining morphology and molecular information are needed to identify these species. *C. fuscocephala* and *C. perexiguus* are mostly distributed in South East Asia and the Mediterranean countries respectively (Walter Reed Biosystematics Unit, www.wrbu.org).

**Figure 3:**
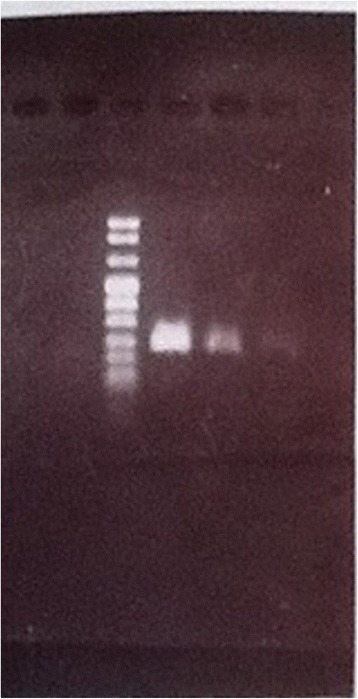
***S*** **pecies diagnostic PCR for** ***Culex decens*** **using the primers ‘cddir and cdrev’.** Lane 1 – 1 kb ladder, lane 2 – *Culex* mosquito from Cape Coast (sequence of mitochondrial cytochrome c oxidase (CO1) gene of this mosquito was close to existing COI of *Culex decens* in Genebank), Lane 3 – *Culex* species from Cape Coast (from the same breeding habitat with the one in lane 2), lane 4 – *Culex quinquefasciatus* from Accra.

PCR diagnostic assay using primers designed from mitochondrial COI gene sequence of *C. decens* confirmed *C. decens* in some of the study sites (Table 2; Figure 3). This is not surprising since *C. decens* is a widely distributed species in Ghana [15,16]; however their presence in polluted breeding habitats are not well known. The collection of *C. decens* in polluted waters is in agreement with Opoku and colleagues [16], who found *C. decens* occurring sympatrically with *C. quinquefasciatus* in polluted habitats in Accra.

**Table 2:** **Distribution of** ***ace*** **1 mutation (G119S) in** ***Culex*** **species from different urban areas in Ghana**

**Study sites**	***Culex*** **species**		***ace*** **1 mutation**			
	**C.q**	**C.d**	**SS**	**RS**	**RR**	**F(R)**
Accra	25	0	48% (12)	40% (10)	12%(3)	0.32
Cape Coast	0	10	100% (20)	0%(0)	0%(0)	0
Kumasi	16	0	81.3% (13)	12.5(2)	6.2%(1)	0.13
Sunyani	0	10	36.4% (4)	45.5% (5)	18.2% (2)	0.41
Techiman	0	5	80% (4)	20%(1)	0%(0)	0.1

Value in bracket represents sample size. C.q – *Culex quinquefasciatus*, C.d – *Culex decens*, SS – homozygote susceptible, RS – heterozygote resistant, RR – homozygote resistant, F(R) – frequency of resistant allele.

Besides *C. quinquefasciatus* and *C. decens*, existence of other *Culex* species in the study sites is possible but the result from this study was unable to identify or confirm the identity of such species. There is a need for further studies on the diversity and habitat ecology of *Culex* species in Ghana. Also the development of molecular tools to identify them is vital since existing molecular tools for the identification of *Culex* species mostly target members of the *C. pipiens* complex. With further evaluation, the primers ‘cddir and cdrev’ could be useful molecular tools in identifying *C. decens*.

### Insecticide susceptibility assay

**Figure 4:**
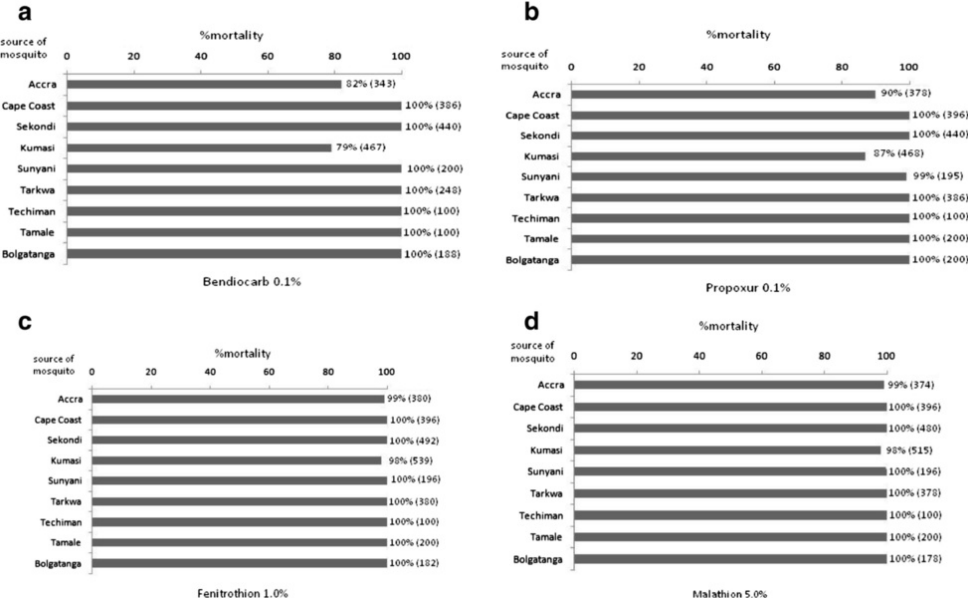
**Resistance status of** ***Culex*** **species to organophosphate and carbamate insecticides in urban areas in Ghana, a) bendiocarb b) propoxur c) fenitrothion d) malathion.**

*Culex* species from the study populations were susceptible to organophosphate (malathion, fenitrothion). Also, the level of carbamate (propoxur, bendiocarb) resistance was generally very low (Table 3; Figure 4). In total the carbamate-induced percentage mortality (± SD) was 94.1% ± 15.4 whereas mortality caused by organophosphate was 99.5% ± 2.2 and the difference between the two mortalities was significant (Mann Whitney U: p < 0.05). Resistance to carbamate was only found in *C. quinquefasciatus* populations from Accra and Kumasi (large urban areas). Complete susceptibility of *Culex* species in Ghana to organophosphate is good for vector control and resistance management. Although, the level of resistance to carbamate observed in this study may not have any serious implication on vector control, there is a need for constant monitoring and resistance surveillance to prevent resistance to these insecticides rising to a level that could affect insecticide resistance management strategies in the country. Environmental variables such as ecology, seasons and land use settings were marginally or non-significantly associated with carbamate and organophosphate resistance (Table 3). *Culex* species in Ghana and most West African countries are not epidemiologically important but many households spent a considerable amount of money and resources to prevent their nuisance [17,18]. Furthermore, the influence of *Culex* species on the use of insecticide treated net has been highlighted by several studies [19,20]. These reasons emphasise the importance of controlling and monitoring insecticide resistance in *Culex* species.

**Table 3:** **Percentage mortality (95% CI) of** ***Culex*** **species to organophosphate and carbamate insecticides and different environmental factors associated with it**

**Environmental factors**		**Mean mortality (%)**			
		**Carbamate**		**Organophosphate**	
		**Bendiocarb** ^*^	**Propoxur** ^*^	**Fenitrothion** ^*^	**Malathion** ^*^
Land use	Residential	87^a^	93^a^	99^a^	99^a^
		(78–95)	(86–99)	(98–100)	(98–100)
	Urban farm	98^a^	99^a^	99^a^	100^b^
		(95–101)	(99–101)	(99–100)	(100–100)
	Swampy	98^a^	97^a^	99^a^	100^b^
		(94–101)	(94–100)	(99–100)	(100–100)
Season	Rainy	89^a^	96^a^	99^a^	99^a^
		(80–97)	(93–99)	(98–100)	(98–100)
	Dry	95.7^a^	96^a^	99^a^	100^a^
		(92–99)	(90–101)	(99–100)	(99–100)
Ecology	Coastal savannah	94^a,b^	97^a,b^	99^a^	100^a^
		(89–100)	(95–100)	(99–100)	(99–100)
	Forest	88^b^	93^b^	99^a^	99^a^
		(80–97)	(86–99)	(98–100)	(98–100)
	Guinea savannah	100^a^	100^a^	100^a^	100^a^
		(100–100)	(100–100)	(100–100)	(100–100)

*In each insecticide, values in columns (environmental factors) sharing same letter are not significantly different.

### Multiple insecticide resistance mechanisms

**Figure 5:**
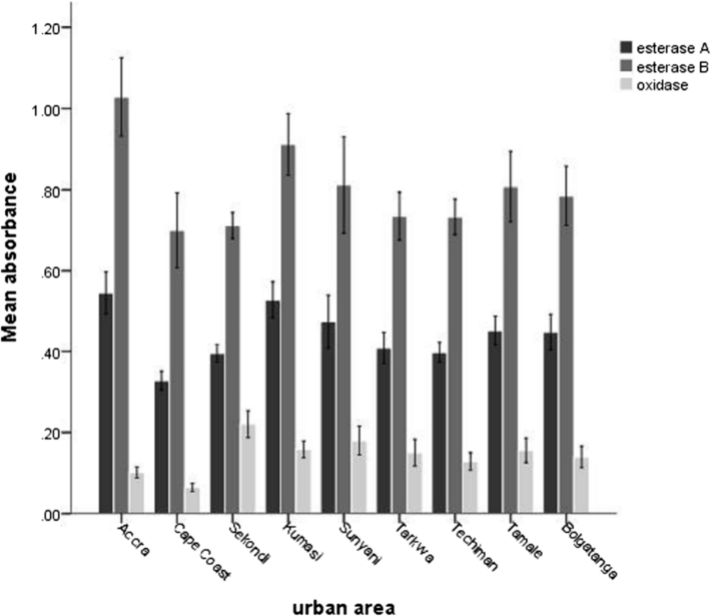
**Mean absorbance (optic density) of α-esterase (esterase A), β-esterase (esterase B) and mixed function oxidase of** ***Culex*** **species from different urban areas in Ghana.** (Error bar: 95% CI).

Overall mean absorbance of α-esterase was 0.448 ± 0.12, β-esterase, 0.807 ± 0.22 and oxidase, 0.152 ± 0.08 (Figure 5). Absorbance of all the three enzymes tested was higher in C*. quinquefasciatus* populations from Accra and Kumasi than the other *Culex* species. A strong negative correlation was observed between percentage mortality from the bioassay and mean absorbance of β-esterase (Pearson r = −0.841, p = 0.004). However, such significant association was not observed between percentage mortality and α-esterase (Pearson r = − 0.45, p = 0.22) or oxidase (Pearson r = 0.131, p = 0.738). This may suggest the involvement of enzyme activity particularly esterases in the resistance of *C. quinquefasciatus* to carbamate insecticides. Enhanced levels or modified activities of esterases and other detoxifying enzymes have been reported to prevent some insecticides from reaching their site of action [21]. Meanwhile, several studies have shown close association between organophosphate and carbamate resistance and high levels of esterases [22,23].

In mosquitoes, insensitive acetyl cholinesterase (AChE) is another common resistance mechanism to organophosphate and carbamate insecticides [24]. A single mutation (G119S of the *ace*1 gene) is responsible for AChE insensitivity to the two classes of insecticides [13]. The PCR diagnostic assay that was performed in the present study detected the *ace*1 mutation (G119S) in some of the mosquito populations (Table 2) especially in *C. quinquefasciatus*. Also, *ace*1 mutation was detected in *C. decens*; however, the frequency of the mutation should be interpreted with caution owing to the small sample size. Approximately equal numbers of mosquitoes (about 20 mosquitoes) from each site were used but most study sites failed to produce results (DNA failed to amplify) with the exception of mosquitoes from Accra and Kumasi. Since this method [13] was originally designed to detect *ace*1 mutation in *Anopheles* species and *C. pipiens* complex to which C*. quinquefasciatus* belongs, it is possible that the method is not sensitive enough for other *Culex* species particularly those found in this study. This may partly explain why most of the DNAs that were not from *C. quinqefasciatus* failed to produce results. Within the two *C. quinquefasciatus* populations, the distribution of the *ace*1 genotypes did not differ from the Hardy-Weinberg equilibrium (*P* > 0.05, Table 2).

Despite *ace*1 mutations being reported to provide cross resistance to organophosphate and carbamate [25], the resistance level greatly varied between the two classes of insecticides. However, some studies have suggested that *ace*1 mutations have a greater impact on carbamate than organophosphate resistance [20,26]. The reason for this is unclear at this time.

### Impact of urbanization on insecticide resistance

**Figure 6:**
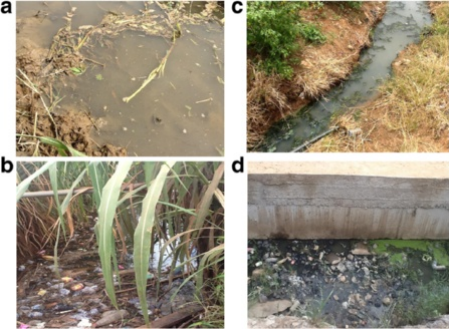
**a-d Polluted breeding habitats where mosquito larvae were collected - a) Tarkwa, b) Sunyani, c) Kumasi, d) Accra.**

The distribution of *Culex* species in this study appears to be influenced by the degree of urbanization. *C. quinquefasciatus* was found in the populous and most urbanized areas in Ghana whereas *C. decens* and other unidentified *Culex* species were found in relatively small urban areas. Although the level of pollution was not quantified in the breeding sites, it was nevertheless observed that breeding sites where mosquitoes were collected were more polluted in Accra and Kumasi than those in other urban areas (Figure 6a-d). It was therefore not surprising that *C. quinquefasciatus* was found in these areas since *C. quinquefasciatus* are most common in polluted heavily urbanized sites. The results also showed that other *Culex* species such as *C. decens* are also breeding in polluted water bodies in urban areas in Ghana.

Resistance to carbamate were observed in *C. quinquefasciatus* populations from Accra and Kumasi (Table 3). In addition, high levels of esterases and *ace*1 mutations (Table 2) were found in the same population. This indicates a higher insecticide selection pressure in Accra and Kumasi (large urban areas) than the rest of the study sites (small urban areas). There are strong arguments in favour of agricultural and domestic use of insecticides as the major cause of resistance in urban areas [27]. Yet, despite the use of organophosphate in urban farms, mosquitoes collected from those areas were susceptible to the insecticides. Additionally, the use of carbamate and organophosphate for malaria control or domestic use of these insecticides for mosquito control is limited. Therefore, agricultural and domestic use of insecticides cannot fully explain the cause of resistance in the present study.

Another cause of resistance in mosquitoes which until recently has received little attention is the presence of pollutants in breeding habitats of mosquitoes. Exposure of mosquitoes to common urban pollutants has been shown to increase mosquito tolerance to insecticides in some studies [28,29]. We suspect pollutants found in breeding habitats to have played a role in resistance of *C. quinquefasciatus* to carbamate in this study. However, the result from the study cannot confirm this hypothesis. In a related study, Chandre and colleagues [30] were also not able to relate carbamate and organophosphate resistance in *C. quinquefasciatus* in Ivory Coast and Burkina Faso to the use of agricultural pesticides but rather implicated domestic use of insecticides to be associated with corresponding resistances. Similarly, *Anopheles* species that were sampled from an urban vegetable farm in Accra were susceptible to organophosphate, though several organophosphate insecticides were detected in the water body in which the mosquito was breeding [31]. On the contrary, Essandoh and colleagues [20] implicated agricultural use of pesticides as a cause of carbamate and organophosphate in *Anopheles* species from peri-urban agricultural area near Accra.

The presence of *C. decens* in polluted breeding sites, which is not known to breed in such habitats, coupled with various reports that have observed *A. gambiae*, a major malaria vector, also breeding in polluted habitats [32,33] reinforces the need to take a critical look at the numerous polluted breeding sites scattered in the country. Both solid and liquid waste are poorly managed in urban areas in Ghana as well as many African countries and what enters into gutters or water bodies from commercial and domestic activities is not much regulated. This presents a situation where apart from pesticides officially sanctioned for public use, mosquitoes could also be exposed to unknown chemicals or insecticides in polluted breeding habitats, which can select for resistance mechanisms that can confer high level of resistance to new insecticides that officially have not been recognized to be used in the country.

## Conclusions

The study found low prevalence of resistance to carbamate and organophosphate insecticides among *Culex* species from Ghana. Due to low levels of resistance to the insecticides, resistance management strategies comprising the use of organophosphate and carbamate may be successful against pyrethroid-resistant *Culex* species in Ghana*.* However, such strategies must be carried out with caution since populations with *ace*1 mutations and high levels of esterases, which can confer high resistance to these classes of insecticides, already exist.

This is the first study in Ghana showing evidence of the existence of multiple insecticide resistance mechanisms in *C. quinquefasciatus* to carbamates and organophosphates. With the existence of populations with multiple resistance mechanisms, it is important to monitor activities or behaviours that have the potential to select for resistance populations. Besides agriculture and domestic use of insecticides, urban pollutants found in mosquito breeding habitats have also been implicated as a cause of resistance. With the expansion of agricultural activities in urban areas, the amount of pesticides used is greatly increasing. In addition, inadequate urban infrastructure, partly due to rapid population growth, leads to creation of numerous polluted habitats that are suitable for mosquito breeding. For these reasons, proper management of waste, particularly in urban areas, and effective regulation of the use of pesticides appear to be critical for managing insecticide resistance.
